# Mitochondrial sirtuin 3 and various cell death modalities

**DOI:** 10.3389/fcell.2022.947357

**Published:** 2022-07-22

**Authors:** Maria A. Yapryntseva, Polina V. Maximchik, Boris Zhivotovsky, Vladimir Gogvadze

**Affiliations:** ^1^ Faculty of Basic Medicine, Lomonosov Moscow State University, Moscow, Russia; ^2^ Karolinska Institutet, Institute of Environmental Medicine, Stockholm, Sweden

**Keywords:** sirtuin, reactive oxygen species, mitochondria, tumor elimination, cell death modalities

## Abstract

Sirtuin 3, a member of the mammalian sirtuin family of proteins, is involved in the regulation of multiple processes in cells. It is a major mitochondrial NAD^+^-dependent deacetylase with a broad range of functions, such as regulation of oxidative stress, reprogramming of tumor cell energy pathways, and metabolic homeostasis. One of the intriguing functions of sirtuin 3 is the regulation of mitochondrial outer membrane permeabilization, a key step in apoptosis initiation/progression. Moreover, sirtuin 3 is involved in the execution of various cell death modalities, which makes sirtuin 3 a possible regulator of crosstalk between them. This review is focused on the role of sirtuin 3 as a target for tumor cell elimination and how mitochondria and reactive oxygen species (ROS) are implicated in this process.

## Introduction

Understanding the mechanisms of cell death is just as important as understanding those of cell division and differentiation because, together, these processes regulate cell homeostasis and tissue renewal in the body. Violations of this balance can cause any number of abnormalities. Thus, an increased level of cell death might be the cause of several autoimmune and neurodegenerative diseases, while the suppression of cell death can stimulate tumor formation. By contrast, stimulation of programmed cell death (PCD) is an effective mechanism of tumor cell elimination. This is one reason why the study of the cell death machinery is one of the most actively developing areas of biomedical research. According to the modern classification, there are more than a dozen types of cell death. Indeed, the phenomenon of PCD, based on the existence of genes encoding proteins implemented the death program, is beyond a doubt.

In the search for targets that regulate the balance between proliferation and cell death, investigators were attracted to sirtuin 3 (Sirt3), a member of the mammalian sirtuin family of proteins (Kincaid and Bossy-Wetzel, 2013). Sirt3 is a major mitochondrial NAD^+^-dependent deacetylase with a broad range of functions. Sirt3 is expressed as a full-length 44 kDa protein, transported to mitochondria, and cleaved within it by matrix metalloprotease to a 28 kDa short form. It is considered that only processed form has deacetylase activity, and it is found only in mitochondria. Although some amount of full-length Sirt3 could exist in the nucleus and cytoplasm, active Sirt3 is localized only inside mitochondria and acts through deacetylation of the mitochondrial proteins (Iwahara et al., 2012). One of these functions, which might be important for both proliferation and cell death, is the regulation of oxidative stress. Reactive oxygen species (ROS), generated largely in mitochondria, at physiological levels, are an important regulator of cellular metabolism. In particular, they facilitate tumor cell proliferation and migration (Kumari et al., 2018), as well as differentiation (Tsatmali et al., 2006), and contribute significantly to the immune response (Yang et al., 2013). It is known that excessive ROS production can lead to cell death either by apoptosis or by necrosis, depending on the severity of oxidative stress. In addition to these well-described cell death modalities, several observations point to the involvement of ROS in other forms of PCD and accompanying processes. ROS has been shown to trigger Parkin/PINK1 pathway-dependent mitophagy, an important mitochondrial quality control mechanism in the cell (Xiao et al., 2017); necroptosis or regulated necrosis (Zhang et al., 2017b); and ferroptosis, a regulated form of cell death driven by the accumulation of lipid peroxidation products (Yang and Stockwell, 2016).

## Sirtuin 3 and reactive oxygen species

**FIGURE 1:**
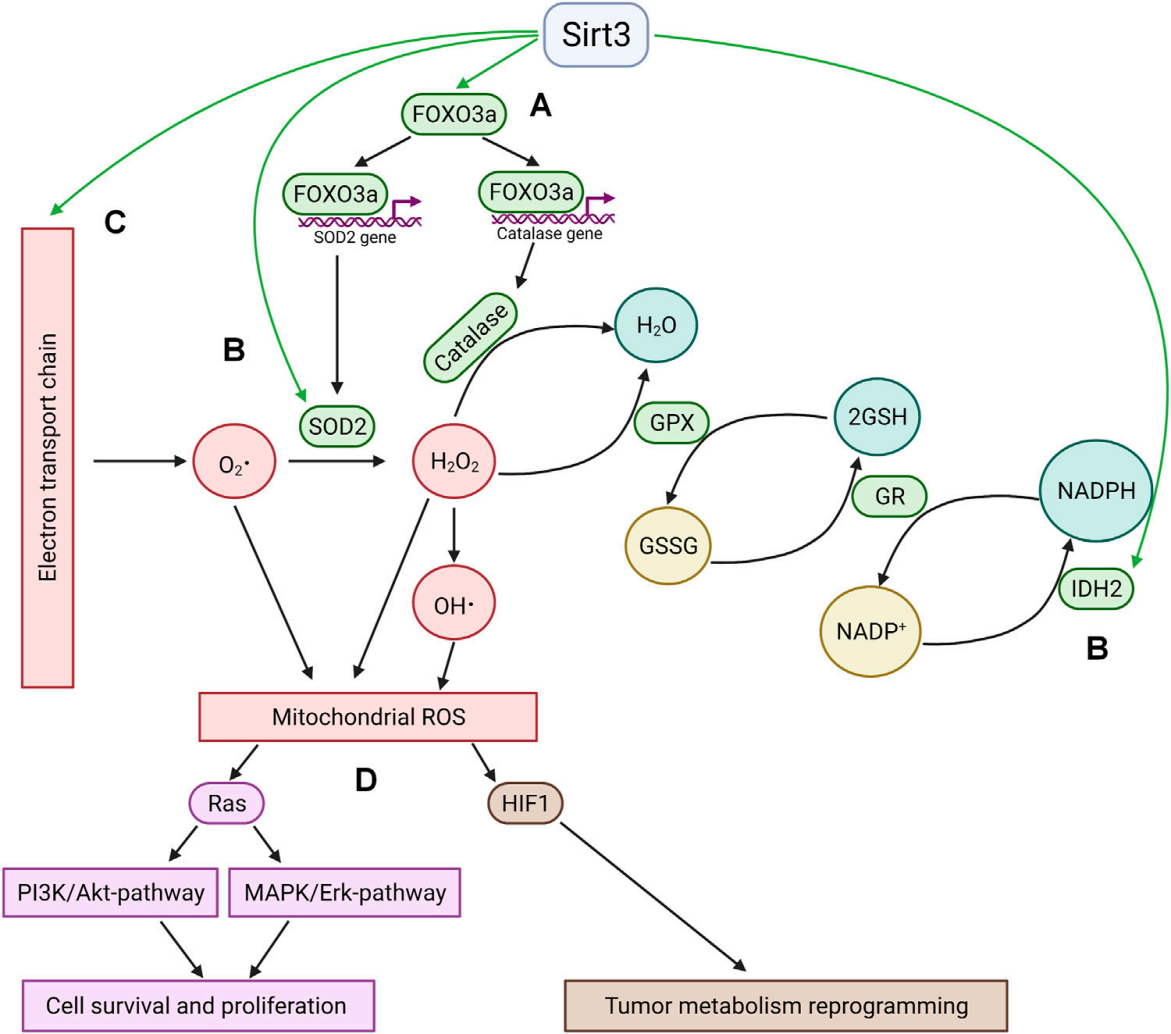
Sirtuin 3 and ROS. **(A)**; Sirt3 stimulates the expression of key antioxidant enzymes MnSOD2 and catalase *via* deacetylation of FOXO3a. Deacetylation occurs either in the mitochondria with subsequent translocation of FOXO3a into the nucleus or by means of nuclear fraction of Sirt3 in the nucleus. **(B)**; Sirt3 directly deacetylates and activates mitochondrial enzymes implicated in ROS quenching (IDH2 and SOD2). **(C)**; Sirt3 decreases electron leakage from mitochondrial electron transport chain by deacetylation of its complexes. **(D)**; The antioxidant activity of Sirt3 leads to HIF1 destabilization and Ras-signaling suppression. GPX—glutathione peroxidase, GR—glutathione reductase, GSSG—oxidized glutathione, GSH—reduced glutathione.

Among the targets of Sirt3 is the transcription factor Forkhead box O3 (FOXO3a), one of the key regulators of cancer cell homeostasis. In metabolically stressed cells, FOXO3A can be recruited to the mitochondria through the activation of MEK/ERK and AMP-activated protein kinase (AMPK) where it stimulates the expression of genes involved in the regulation of mitochondrial metabolism (Celestini et al., 2018). Besides, several observations demonstrate that deacetylated FOXO3a controls the expression of nuclear antioxidant-encoding genes, including manganese superoxide dismutase (MnSOD) and catalase (Sundaresan et al., 2009; Tseng et al., 2013). FOXO3a deacetylation can promote its nuclear localization (Wang et al., 2017), however the mechanism of its release from the mitochondria remains obscure. On the other hand, FOXO3a can be deacetylated by a small fraction of nuclear Sirt3 (Scher et al., 2007; Iwahara et al., 2012). Sirt3 can also directly deacetylate several specific lysine residues and thus directly activate MnSOD (Tao et al., 2014) and isocitrate dehydrogenase 2 (IDH2) (Yu et al., 2012). IDH2, a key generator of NADPH in mitochondria, is critical for the maintenance of mitochondrial redox balance (Reitman and Yan, 2010) and is involved in glutathione reduction. Sirt3 can also directly deacetylate mitochondrial electron-transport chain complexes, decreasing electron leakage and ROS production (Rahman et al., 2014). Increased Sirt3 expression attenuates ROS accumulation in the cell (Figure 1).

The antioxidant activity of Sirt3 modulates different signaling pathways involved in cell death and survival. Thus, a Sirt3-driven decrease in ROS levels suppressed Ras activation and downstream signaling through the MAPK/ERK and PI3K/Akt pathways, which activate cell survival and proliferation (Sundaresan et al., 2009). Another significant consequence of Sirt3-mediated inhibition of ROS production is the downregulation of hypoxia inducible factor 1 (HIF1), which plays a key role in the regulation of tumor cell metabolism (Bell et al., 2011; Finley et al., 2011; Geoghegan et al., 2017). This factor can be stabilized under hypoxia, as well as by some mitochondrial substrates (succinate and fumarate, a phenomenon known as pseudohypoxia), or by ROS (Chandel et al., 2000).

## Pro- and antitumor effects of sirtuin 3

Sirt3 can function as an oncogene and support cancer cell proliferation. Some recent studies show that Sirt3 can deacetylate the tumor suppressor transcription factor p53, which in turn regulates dozens of target genes with diverse biological functions. This deacetylation was shown to lead to p53 degradation, which facilitated tumor cell proliferation (Xiong et al., 2018). The authors did not explain the mechanism of deacetylation of p53 by mitochondrially located Sirt3. Their conclusion was based on the data showing that acetylation of p53 decreased upon overexpression of Sirt3. According to another point of view, there was no evidence showing that Sirt3 can deacetylate p53. In hepatocellular carcinoma cells, Sirt3 overexpression upregulated p53 protein level without altering p53 mRNA, suggesting that Sirt3 may modulate p53 *via* post-transcriptional regulation (Zhang and Zhou, 2012). The authors proposed that the Sirt3 overexpression induced downregulation of Mdm2, which targets p53 for proteasomal degradation, thereby increasing p53 protein level. On the other hand, deacetylation of p53 by Sirt3, which stimulated cell proliferation and prevented p53-mediated growth arrest, was documented in bladder cancer. However, it has been stated that deacetylation by Sirt3 was restricted only to mitochondrial p53 (Li et al., 2010). Sirt3 can promote tumorigenesis through its antioxidant activity. Thus, the Sirt3/FOXO3a/SOD2 axis of the mitochondrial unfolded protein response (UPRmt) was activated in cancer cells in response to oxidative proteotoxic stress in mitochondria, increasing mitochondrial fitness and supporting metastasis (Kenny et al., 2017). The ability of Sirt3 to maintain ROS production at the appropriate levels prevents apoptosis stimulation and facilitates cell proliferation (Kim et al., 2011). Suppression of Sirt3 expression in breast cancer cells, and the subsequently elevated ROS production, makes these cells more susceptible to anticancer drugs (Torrens-Mas et al., 2017).

On the other hand, a number of observations indicate that Sirt3 has antitumor effects (Zeng et al., 2017). Thus, Sirt3 knockout mice may spontaneously develop breast tumors (Kim et al., 2010). Murine models that lack Sirt3 are characterized by increased malignancies that resemble human luminal B breast cancer. Furthermore, these tumors exhibit aberrant acetylation of MnSOD at lysine 68 and lysine 122 and have abnormally high ROS levels (Zou et al., 2016). Stimulation of Sirt3 expression in these cells normalizes the level of oxygen radicals. As mentioned above, one of the pathways by which Sirt3 controls tumor growth is destabilization of HIF1. Sirt3 modulates HIF1 level through ROS-mediated alteration of proline hydroxylation enzyme function, thereby altering hydroxylation and subsequent proteasomal degradation of HIF1α. As it has been shown, cancer cells rely on glycolysis even in the presence of oxygen, a phenomenon known as the Warburg effect (Warburg and Dickens, 1930). Such a glycolytic shift provides rapidly proliferating tumor cells with the substrates necessary for biomass generation. The main regulatory factor in this process is HIF1 (Semenza, 2012). This protein stimulates the expression of a myriad of target genes involved in glycolysis regulation and angiogenesis. Reduction of Sirt3 expression occurs in human breast tumors, and its loss correlates with the activation of HIF1 target genes (Finley et al., 2011). Sirt3 suppression in primary mouse embryo fibroblasts (MEFs) or tumor cell lines stimulates cell proliferation and augments HIF1α protein stabilization and its transcriptional activity under hypoxic conditions. Conversely, Sirt3 overexpression prevents HIF1α stabilization and attenuates the elevation of HIF1α transcriptional activity. Sirt3 knockdown also increases tumorigenesis in xenograft models, while its overexpression decreases tumor formation (Bell et al., 2011). Sirt3 can deacetylate and thus stimulate mitochondrial pyruvate dehydrogenase. This intensifies mitochondrial activity, and reverses the glycolytic shift in tumor cells (Fan et al., 2014). Thus, increased Sirt3 expression attenuates glycolysis and cancer cell proliferation, both of which represent a metabolic mechanism for tumor suppression. Apparently, the consequences of this Sirt3 targeting are dependent on the specific cell’s reliance on glycolysis. It can be assumed that this Sirt3 property will be particularly important in the treatment of tumors with the most pronounced glycolytic shift.

## Sirtuin 3 and various cell death modalities

### Sirtuin 3 and apoptosis

Mitochondria play a decisive role in apoptosis induction/progression. Outer mitochondrial membrane (OMM) permeabilization, and the subsequent release of pro-apoptotic factors such as cytochrome *c* and apoptosis inducing factor (AIF), activates a cascade of reactions that execute apoptotic cell death. Thus, apoptosis can be regulated at the level of the mitochondria, by modulation of the processes that precede permeabilization, and by downstream processes. Accordingly, the resistance of cells to chemotherapy may be due to disturbances that occur at these levels. Understanding the mechanism of apoptosis resistance helps to identify ways to modulate emerging disorders and eliminate them. Additionally, the use of substances that act on the mechanism(s) responsible for resistance to apoptosis, in combination with the recommended chemotherapy, should increase the effectiveness of treatment and, preferably, reduce the dose of chemotherapeutic agents to reduce side effects.

**FIGURE 2:**
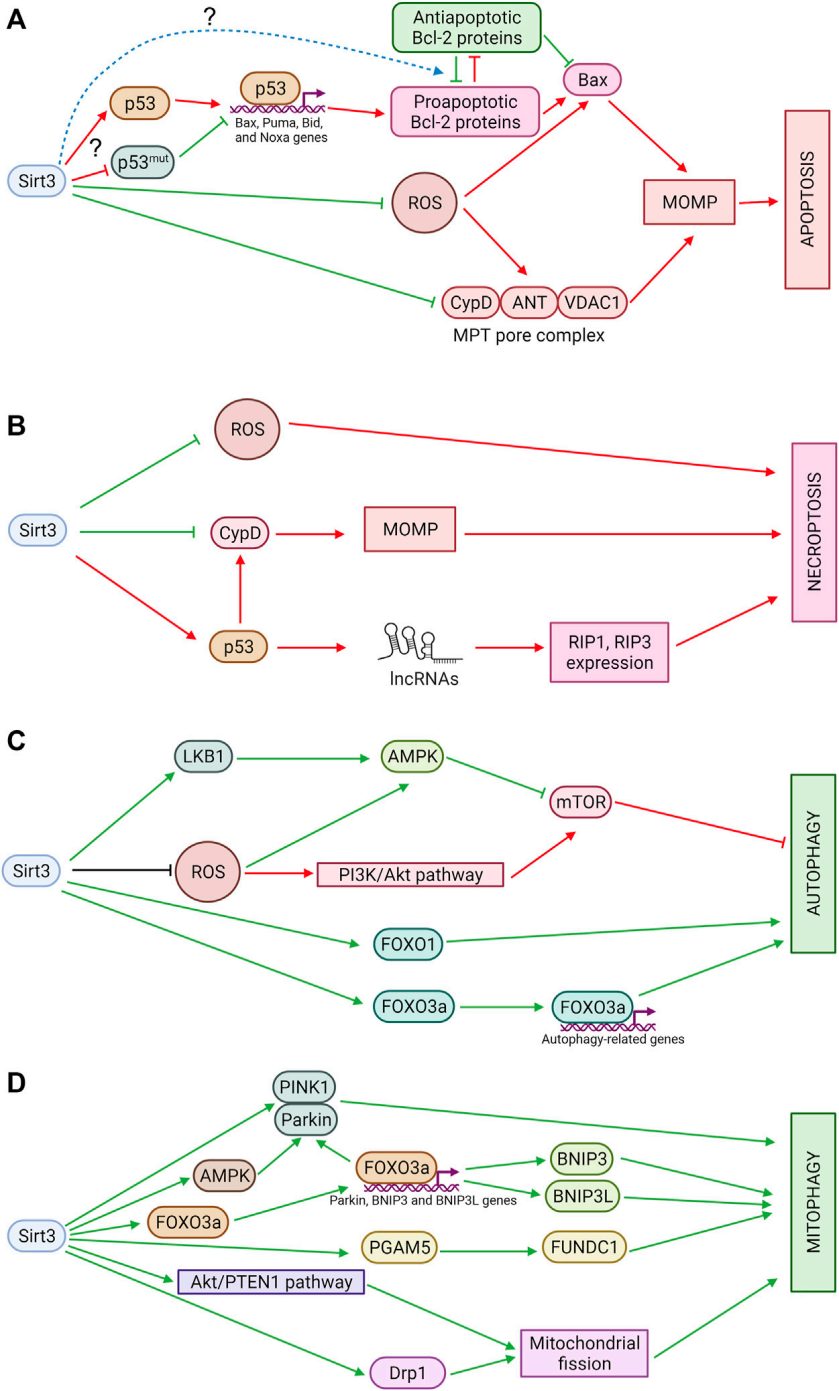
**(A)** Sirtuin 3 and apoptosis. Sirt3 suppresses excessive production of ROS, which can stimulate OMM permeabilization through oxidation of thiols in ANT or Bax. In addition, Sirt3 deacetylates CypD, preventing pore opening, and modulates OMM permeabilization mediated by Bcl-2 family proteins, although the mechanisms of these processes are still poorly understood. Red arrows denote pro-apoptotic effects and green arrows denote anti-apoptotic effects. **(B)** Sirtuin 3 and necroptosis. Sirt3 inhibits mitochondrial ROS production and prevents MPT pore opening which can be involved in necroptosis. Sirt3 also deacetylates p53 which promotes necroptosis in mitochondria-independent way. LncRNAs—long non-coding RNAs. Red arrows denote pro-necroptotic effects and green arrows denote anti-necroptotic effects. **(C)** Sirtuin 3 and autophagy. Sirt3 promotes autophagy *via* AMPK and FOXO1 activation. It also stimulates expression of autophagy-related genes *via* FOXO3a deacetylation. Sirt3 can inhibit autophagy *via* suppression of ROS-dependent AMPK-pathway stimulation and *via* acetyl-CoA pools maintaining. Green arrows denote pro-autophagic effects and red arrows denote anti-autophagic effects. **(D)** Sirtuin 3 and mitophagy. Sirt3 stimulates PINK1/Parkin-dependent as well as receptor-mediated mitophagy. Sirt3 also promotes mitochondrial fission which is a prerequisite of mitophagy. Green arrows denote pro-mitophagic effects.

There are two main OMM permeabilization pathways. One is regulated by the balance between pro- and anti-apoptotic Bcl-2 family proteins. Oligomerization of pro-apoptotic proteins (Bax, Bak) and their subsequent incorporation into OMM forms a pore through which proapoptotic factors can be released into the cytosol. Anti-apoptotic members of the Bcl-2 family prevent pore formation through binding to pro-apoptotic proteins. The expression of several proapoptotic Bcl-2 family proteins, including Bax, Puma, Bid, and Noxa, is regulated by p53 (Figure 2A).

Data on the relationship between Sirt3 and proteins of the Bcl-2 family are to some extent controversial. Sirt 3 was shown to activate p53 and induce apoptosis in hepatocellular carcinoma (Zhang and Zhou, 2012). In lung adenocarcinoma cells, Sirt3 overexpression augmented the Bax/Bcl-2 and Bad/Bcl-XL ratios and promoted AIF translocation to the nucleus while the level of p53 was increased. The mechanisms that underlie these changes can involve transcriptional activation of Bax by p53; however, for now they remain unclear (Xiao et al., 2013). In many cancers, p53 is mutated. Mutant p53 acts in the opposite way to normal p53, inhibiting its apoptotic functions. In small-cell lung cancer cells, Sirt3 was shown to promote mutant p53 deacetylation and its proteasomal degradation. Sirt3 overexpression also increased the Bax/Bcl-2 ratio and induced apoptosis (Tang et al., 2019; Guo et al., 2020). Other Bcl-2 proteins are also regulated by Sirt3; thus, in NCI-H446 human small-cell lung cancer cells, the proapoptotic Bid level was elevated and the antiapoptotic Mcl-1 level decreased (Tang et al., 2019). Sirt3 can also directly deacetylate antiapoptotic Mcl-1, inhibiting its interaction with ubiquitin-specific peptidase 9 X-linked (USP9X), resulting in Mcl-1 phosphorylation, ubiquitination, and destabilization. Sirt3 depletion leads to cell death resistance (Shimizu et al., 2021). In HCT116 cells, Sirt3 also revealed pro-apoptotic properties that were linked to Bcl-2/p53 regulation of apoptosis, as Sirt3 silencing prevented apoptosis induced by loss of Bcl-2 (Allison and Milner, 2007). It was also shown that Sirt3 deacetylated and activated glycogen synthase kinase-3β (GSK-3β), which subsequently induced expression and mitochondrial translocation of Bax, inducing apoptosis (Song et al., 2016).

On the other hand, Sirt3 downregulation in esophageal squamous cell carcinoma EC9706 cells increased the expression of p21 and Bax proteins but reduced Bcl-2 protein expression, significantly inhibited cell proliferation, and induced apoptosis (Yang et al., 2014). Sirt3-mediated deacetylation of Ku70 in human glioma cells stabilized the Ku70/Bax interaction and made cells more resistant to Bax-mediated apoptosis (Luo et al., 2018). Sirt3 overexpression in MDA-MB-231 human breast carcinoma cells prevented Bax accumulation induced by staurosporine (Pellegrini et al., 2012). Cardiomyocytes subjected to ischemia-reperfusion after curcumin treatment demonstrated activation of Sirt3, Bcl-2 upregulation, and Bax downregulation, which decreased apoptosis. Sirt3 inhibition reversed its protective effect (Wang et al., 2018b).

Additionally, OMM permeabilization may result from mitochondrial swelling, which causes ruptures in the OMM and subsequent release of proapoptotic factors from the intermembrane space. This release is due to the opening of a non-specific pore, the mitochondrial permeability transition (MPT) pore, which occurs in the inner membrane in response to stressful effects. Pore opening leads to the entry of water and solutes in the mitochondrial matrix causing mitochondrial swelling, the rupture of OMM and the release of proteins from intermembrane space. Stimulation of the opening of a nonspecific pore underlies various pathologies, in particular, ischemic disease, while the increased resistance of the mitochondrial membrane to permeabilization, which suppresses apoptosis, can stimulate uncontrolled tumor cell growth. The key structural components of the pore, according to the classical scheme, are the adenine nucleotide translocase (ANT) in the inner mitochondrial membrane (IMM) and the voltage-dependent anion channel (VDAC) in the OMM. The main critical regulator of pore opening is cyclophilin D (CypD), a mitochondrial matrix peptidyl-prolyl cis-trans isomerase (Javadov and Kuznetsov, 2013). These proteins catalyze the cis-trans isomerization of peptidyl-prolyl bonds and have chaperone activity to regulate protein folding. Pore opening is attributed to direct binding of CypD to ANT.

Sirt3 can modulate OMM permeabilization *via* deacetylation of proteins that regulate pore formation. One of the Sirt3 targets is CypD, the deacetylation of which diminishes its peptidyl-prolyl cis-trans isomerase activity and causes its dissociation from ANT, contributing to an increase in the MPT pore-opening threshold and preventing its opening (Teodoro et al., 2018). Consistently, cardiac myocytes from mice that lack Sirt3 demonstrated age-dependent mitochondrial swelling due to an increased probability of MPT pore opening (Hafner et al., 2010). Sirt3 activation caused CypD deacetylation and a decrease in the activity attenuating neuron apoptosis in mice with sepsis-associated encephalopathy (Sun et al., 2017). Sirt3-mediated protection against hypoxia or staurosporine can be explained by inhibition of MPT *via* prevention of hexokinase (HKII) binding to the mitochondria (Pellegrini et al., 2012). Sirt3-induced CypD inactivation resulted in the detachment of mitochondrial HKII and inhibition of glycolysis (Wei et al., 2013). Apparently, the protective effect of Sirt3 will be most pronounced in cells in which OMM permeabilization occurs through the opening of non-specific MPT pores.

ROS modulates both types of OMM permeabilization. ROS modifies two thiol groups on ANT, and this modification facilitates pore opening (Halestrap et al., 1997). Furthermore, ROS can mediate the formation of disulfide bridges between cytosolic Bax monomers to promote the formation of pores in the OMM (Neuzil et al., 2006). Depending on the concentration, the same therapeutic compound can stimulate distinct modes of OMM permeabilization, either mediated by pro-apoptotic proteins of the Bcl-2 family or due to the induction of MPT, at least in a certain subpopulation of mitochondria (Robertson et al., 2000). Thus, by controlling ROS levels, Sirt3 can protect cells from stressful effects that result in OMM permeabilization. Sirt3 was shown to have antiapoptotic properties in cardiomyocytes *via* IDH2 deacetylation and ROS quenching (Ma et al., 2021). Sirt3 knockdown sensitized colon cancer cells to anticancer agents by suppressing SOD2 and increasing mitochondrially produced ROS (Paku et al., 2021). Stimulation of Sirt3 expression prevents mitochondrial dysfunction and neurological damage after traumatic brain injury (Wang et al., 2016) and reduces smooth muscle cells apoptosis (Qiu et al., 2021). Interestingly, Sirt3 can regulate mitochondrial function by deacetylating key Krebs cycle enzymes, in particular, succinate dehydrogenase, a subunit of the respiratory chain complex, and thereby stabilize mitochondrial function (Ahn et al., 2008; Finley et al., 2011). Thus, the involvement of Sirt3 in apoptotic cell death execution depends on multiple factors, which can either stimulate or diminish apoptosis upon targeting Sirt3.

### Sirtuin 3 and necroptosis

Necroptosis is a form of regulated cell death that is morphologically similar to necrosis and depends on receptor interacting protein 3 (RIP3) and subsequent mixed lineage kinase domain-like pseudokinase (MLKL) activation. RIP3 is activated by RIP1 when caspase-8 is inactivated (Galluzzi et al., 2018).

Presumably, mitochondria play a pivotal role in necroptosis signaling *via* two major pathways. First, mitochondrial ROS was shown to be critical for necroptosis execution. Sirt3, as an important mitochondrial antioxidant regulator, can control necroptosis (Figure 2B). Consistent with these considerations, in a mouse model of diabetic skin wound healing, Sirt3 deficiency was shown to increase the expression of RIP3K, RIP1, and caspase-3 *via* superoxide production, which results in delayed wound healing, a decreased blood supply, and exacerbated ultrastructural skin disorders. It could be caused by mitochondrial function impairment, oxidative stress, and necroptosis (Du et al., 2017). Sirt3 knockdown promoted oxidative stress and necroptosis in cardiac fibroblasts in hypoxia (Yang et al., 2020). ROS accumulation and necroptosis are correlated with the immune response and have proinflammatory functions. Thus, in prostate cancer, Sirt3 inhibits RIPK3-mediated necroptosis and the innate immune response, promoting tumor progression (Fu et al., 2020).

The other means of mitochondrial involvement in necroptosis is MPT pore induction involving its key regulator, CypD. As was mentioned above, Sirt3 was shown to decrease CypD-mediated MPT pore opening, preventing necroptosis (Sun et al., 2017).

However, it should be mentioned that accumulated data do not confirm the crucial role of mitochondria in necroptosis. Thus, cells depleted of mitochondria through forced mitophagy can undergo necroptosis anyway (Tait et al., 2013). At least, mitochondria are not essential for necroptotic cell death in many types of cells, and their involvement could be context-specific (Marshall and Baines, 2014). Apparently, Sirt3 in necroptosis could be rather dispensable.

Another regulator of necroptotic cell death is p53. It can modulate necroptosis *via* the mitochondrial pathway by binding to CypD. As well, p53 can act independently of mitochondria, transcriptionally upregulating a long-noncoding RNA, which elevates RIP1 and RIP3 expression (Ranjan and Iwakuma, 2016). Sirt3-mediated modulation of p53 may fulfill an interplay between apoptosis and necroptosis. Thus, Sirt3 can directly deacetylate p53, causing its proteasomal degradation and leading to both apoptosis and necroptosis induction in small-cell lung cancer (Tang et al., 2019).

Another cell death type that depends on RIP is anoikis, which is initiated by the loss of extracellular matrix contacts, preventing anchorage-independent proliferation and attachment to an improper matrix. Accordingly, tumor cells need to evade anoikis for metastasis. Sirt3 and RIP regulate overcoming of anoikis in opposite ways. Thus, in oral squamous cell carcinoma, Sirt3 promotes anoikis resistance, whereas RIP inhibits it. It was speculated that the mechanism by which Sirt3 and RIP have opposite functions in anoikis could be mediated by different impacts on CypD and mitochondrial nonspecific pore opening (Kamarajan et al., 2012). Consistently with these data, Sirt3 and SOD2 activity were required for anoikis resistance and anchorage-independent survival and metastasis of ovarian cancer cells (Kim et al., 2020).

### Sirtuin 3 and autophagy

Autophagy is a process in which defective or unnecessary cellular organelles and proteins are delivered to autophagosomes and degraded upon autophagosome fusion with lysosomes. Extensive autophagy may lead to autophagic cell death; however, it usually serves as a cytoprotective process by maintaining cellular homeostasis and recycling cytoplasmic contents (Jung et al., 2020). Autophagy is implicated in crosstalk with other cell death modalities.

The influence of Sirt3 on autophagy is mediated by the energy sensor AMPK, which inhibits the key autophagy suppressor the mammalian target of rapamycin also referred to the mechanistic target of rapamycin (mTOR) and mediates the switch between autophagy and apoptosis (Liang et al., 2007) (Figure 2C). There are data that Sirt3 activates AMPK *via* LKB1 deacetylation (Pillai et al., 2010) and thus promotes autophagy in hepatocytes (Zhang et al., 2020b; Liu et al., 2021a), adipocytes (Zhang et al., 2020a), neurons (Dai et al., 2017), and renal epithelial cells (Tan et al., 2021). Interestingly, Sirt3 also can negatively regulate autophagy in the liver through ROS-mediated AMPK-mTORC1-autophagy pathway suppression and downregulation of MAP1LC3 expression (Li et al., 2017). However, novel data demonstrate that AMPK can act as an upstream Sirt3 regulator (Hu and Ma, 2021; Wang et al., 2021; Li et al., 2022). Additionally, Sirt3 can influence mTOR *via* the PI3K/Akt pathway. *Via* its inhibition, Sirt3 acts as an autophagy promotor (Xu et al., 2021). For this reason, Sirt3 can produce opposing effects on autophagy in liver pathologies, as shown in different studies, and resolution of the mechanism will require further study (Cho et al., 2017).

Sirt3 can deacetylate FOXO1 and then facilitate downstream E3 ubiquitin ligase induction of autophagosome formation (Li et al., 2016). Another Sirt3 target is FOXO3a, which can regulate the expression of autophagy cargo receptor p62. The Sirt3/FOXO3a pathway was reported to promote autophagy in liraglutide-treated mice with non-alcoholic fatty liver disease (Tong et al., 2016) and in a rat model of exsanguinating cardiac arrest treated by emergency preservation and resuscitation (Ma et al., 2019). Autophagy upregulation, elevated MAP1LC3-II and lowered p62/SQSTM1 expression were also associated with Sirt3 signaling in diabetic mice with cardiomyopathy (Zhang et al., 2017a). It seems that involvement of Sirt3 in autophagy is important for the development of various metabolic disorders.

Autophagy plays a key role in the control of cellular redox balance and can be stimulated in response to ROS by the PI3K–Akt and AMPK pathways acting directly on components of the autophagic machinery, such as Atg4 and Beclin-1 (Li et al., 2011). As Sirt3 is one of the main regulators of mitochondrial ROS, it can also control autophagy. It has been demonstrated using a model of cadmium (Cd)-induced autophagic cell death (Pi et al., 2015). Thus, in hepatocytes, Cd decreased the Sirt3 level and suppressed SOD2 deacetylation, and hence its activity, leading to increased ROS production and autophagic cell death. Intriguingly, melatonin suppressed Cd-induced cell death by promoting Sirt3 activity without altering its expression. Similar results were obtained in the A549 cell line, where Cd elevated the ROS level and subsequently promoted MAP1LC3-II, Beclin-1, and Atg4 expression and autophagosome formation (Lv et al., 2018). Another piece of evidence of Sirt3/mtROS-dependent autophagy was presented in human cervical cancer cells treated with metformin in combination with nelfinavir (Xia et al., 2019).

Highly intriguing results about the interplay between Sirt3 function and autophagy in B cell lymphomagenesis were published by Ari Melnick’s group (Li et al., 2019). According to this paper, Sirt3 depletion was followed by the acetylation of glutamine dehydrogenase (GDH) and ensuing suppression of its activity as well as a significant reduction in acetyl-CoA pools with simultaneous induction of autophagic death of tumor cells. This points to Sirt3 as a possible target for the treatment of lymphoma. Moreover, the authors introduced a new mitochondria-targeted class I sirtuin inhibitor, YC8-02, a small-molecule sirtuin inhibitor JH-T4 conjugated with a lipophilic cation triphenylphosphonium, which increases its target specificity for mitochondrial Sirt3.

Besides the role of Sirt3 in carcinogenesis, its essential function in macroautophagy/autophagy regulation in the context of innate immune defense has been established. Sirt3^−/−^ mice were characterized by aberrant mitochondria in tuberculosis-infected cells and macrophages, a massive lung inflammatory response, and increased susceptibility of mice to *Mycobacterium tuberculosis*. It was shown that the peroxisome proliferator activated receptor alpha (PPARA)-transcription factor EB (TFEB) axis acts downstream of Sirt3 signaling and is pivotal in promoting antibacterial autophagy and normal mitochondrial functioning (Li et al., 2019).

### Sirtuin 3 and mitophagy

Mitophagy, the specific autophagic elimination of damaged mitochondria, represents mitochondrial quality control in cells. In particular, removal of mitochondria with permeabilized OMM (proapoptotic mitochondria) can suppress apoptosis (Denisenko et al., 2021). The major mitophagy pathway is initiated by a decrease in mitochondria membrane potential, accumulation of PINK1 on the OMM, and Parkin phosphorylation. Phosphorylated Parkin induces ubiquitination of various OMM proteins and recruitment of mitochondria to autophagosomes. Sirt3 can stimulate mitophagy *via* direct deacetylation of PINK1 and Parkin (Wei et al., 2017) (Figure 2D). It can also increase Parkin expression and activate mitophagy *via* FOXO3 deacetylation (Das et al., 2014; Yu et al., 2017; Hu et al., 2019). Sirt3 may also stimulate Parkin-dependent mitophagy through AMPK activation (Huang et al., 2019). In Sirt3 knockout mice, Sirt3 deficiency impairs Parkin-mediated mitophagy by increasing p53-Parkin binding and blocking the mitochondrial translocation of Parkin in cardiomyocytes (Li et al., 2018b).

In human glioma cells, Sirt3 activated hypoxia-induced mitophagy by increasing the interaction of VDAC1 with Parkin. It prevented proteasomal degradation of the anti-apoptotic proteins Mcl-1 and survivin, thus inhibiting apoptosis and increasing the resistance of tumor cells to hypoxia (Qiao et al., 2016). Sirt3 was shown to prevent a decrease in mitochondrial membrane potential in Cr(VI)-transformed human bronchial epithelial cells and to elevate the expression of PINK1 and Parkin, while Parkin remained in the cytoplasm. This suppressed mitophagy, promoting malignant cell proliferation and tumorigenesis (Clementino et al., 2019). Sirt3 activation by the small-molecule compound 33c (ADTL-SA1215) induced mitophagy and inhibited proliferation and migration in human breast carcinoma cells (Zhang et al., 2021a). The Nrf2/Sirt3 pathway promoted mitophagy, preventing nucleus pulpous apoptosis during intervertebral disc degeneration (Hu et al., 2021). These studies show that Sirt3-mediated mitophagy is involved in apoptosis suppression and regulates cell survival and death.

Another process closely related to mitochondrial quality control is mitochondrial dynamics. Thus, mitochondrial fission is a prerequisite of mitophagy, separating damaged mitochondria from the healthy mitochondrial network (Ikeda et al., 2015). Sirt3 was shown to promote mitochondrial fission *via* the Akt/PTEN pathway, supporting the growth and survival of colorectal cancer cells (Wang et al., 2018c). Modulation of the mitochondrial fission protein Drp1 by Sirt3 could induce mitophagy, protecting cells during ischemia-reperfusion injury (Bai et al., 2021; Zhao et al., 2021).

Besides PINK1/Parkin-dependent mitophagy, receptor-mediated mitophagy plays an important role in mitochondrial quality control. Mitophagy receptors (BNIP3, BNIP3L, also known as NIX, FUNDC1, FKBP8, and Bcl2-L-13) can interact directly with mammalian ATG8 family proteins, targeting mitochondria to autophagosomes. Deacetylation of mitochondrial proteins by Sirt3 initiated mitochondrial autophagy in a Parkin-independent pathway (Webster et al., 2013). Thus, mitophagy receptors can be implicated in this process.

There are a few examples devoted to the interplay between Sirt3 and the major mitophagy receptors BNIP3 and its homologue BNIP3L. Sirt3 was shown to positively regulate BNIP3 and BNIP3L *via* FOXO3 deacetylation, inducing mitophagy in response to oxidative stress to improve mitochondrial quantity and quality (Tseng et al., 2013; Wu et al., 2020). Sirt3 activation by honokiol increases both BNIP3 and BNIP3L levels in rat nucleus pulposus cells in the pathogenesis of an intervertebral disc degeneration model (Wang et al., 2018b). Sirt3 can activate BNIP3-dependent mitophagy *via* the ERK-CREB signaling pathway, protecting hepatocytes in non-alcoholic fatty liver disease (Li et al., 2018a). Sirt3 was shown to induce mitophagy during the unfolded protein response. It proceeded in a Parkin-independent manner, while the BNIP3L level was elevated. These data indicate that Sirt3 might stimulate receptor-mediated mitophagy to maintain mitochondrial integrity (Papa and Germain, 2014).

The opposite effects were shown in diabetic kidney disease, where stanniocalcin-1 activated the AMPK-Sirt3 pathway, thus decreasing ROS production and inhibiting BNIP3 expression. Stanniocalcin-1 and its downstream effects protected kidney cells from apoptosis (Liu et al., 2019). Sirt3 also prevented an increase in the BNIP3 level after doxorubicin treatment, ameliorating mitochondrial dysfunction and protecting cardiomyocytes (Du et al., 2017). Besides functioning as a mitophagy receptor, BNIP3 also has a BH3 domain and can display a weak pro-apoptotic function. Neither of these works touched on mitophagy regulation, investigating only apoptotic functions of BNIP3.

After Cd intoxication in hepatocytes, mRNA levels of Sirt3 and BNIP3 changed in the reciprocal directions. It was speculated that Cd caused mitochondrial disfunction, decreased Sirt3, and increased oxidative stress, which led to activation of BNIP3-dependent mitophagy as well as PINK1/Parkin-dependent mitophagy (Zhang et al., 2021b). It was observed that the level of another mitophagy receptor—FundC1—could also be elevated, together with a decrease in the Sirt3 mRNA level after Cd and Mo intoxication (Wu et al., 2022). However, a decline in Sirt3 could only be a consequence of the accumulation of damaged mitochondria and not cause mitophagy on its own. Neither of these articles reveal the functional link between Sirt3 and mitophagy. Describing the influence of Sirt3 on FundC1, it is worth mentioning that Sirt3 was shown to interact with and deacetylate PGAM5, which phosphorylates FUNDC1 at serine 13 (Ser13). This introduces the Sirt3/PGAM5/FundC1 axis of mitophagy activation (Ma et al., 2017). Apparently, Sirt3 stimulates PINK1/Parkin-dependent mitophagy, as well as receptor-mediated mitophagy, preventing apoptosis.

### Sirtuin 3 and ferroptosis

**FIGURE 3:**
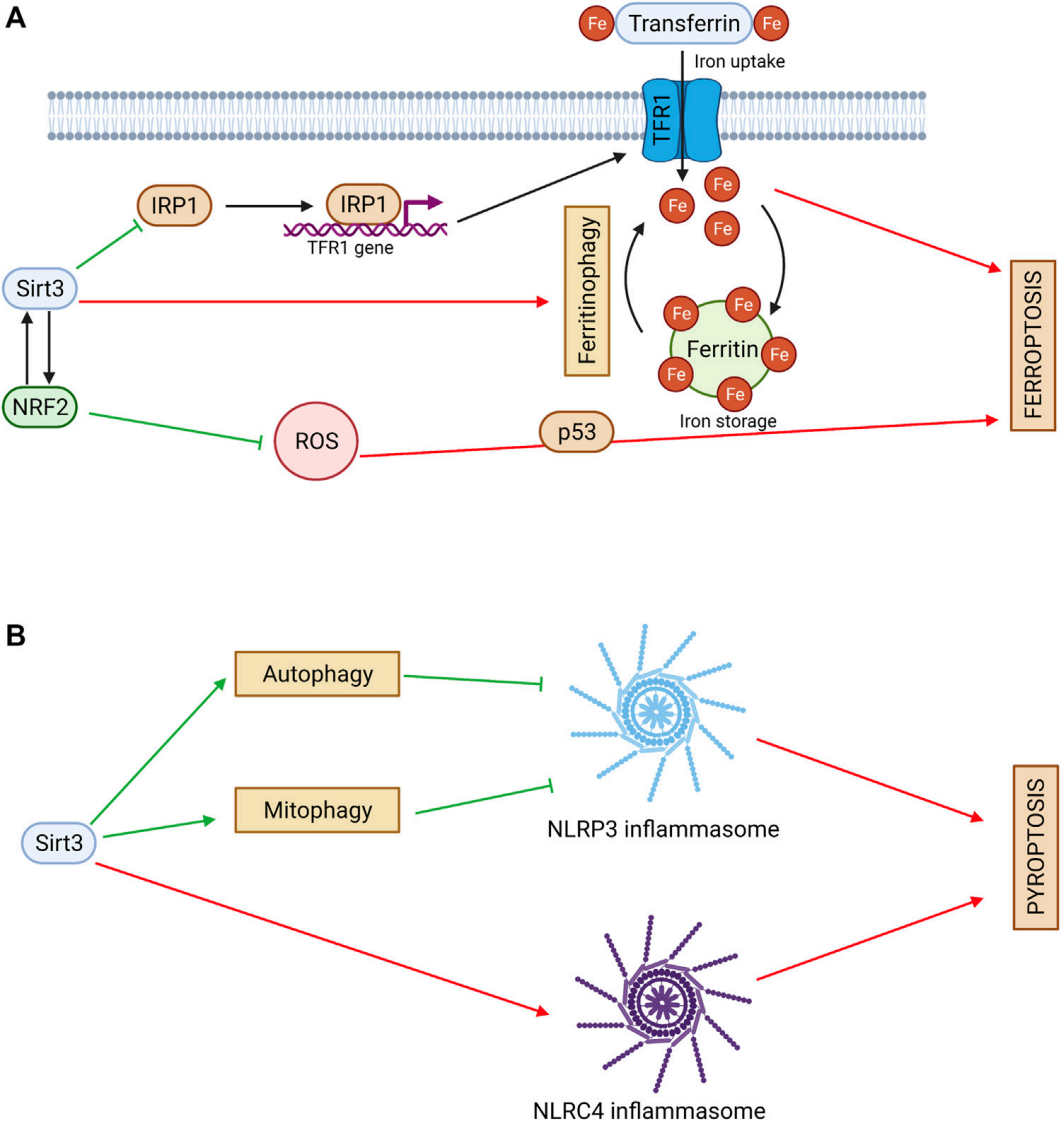
**(A)** Sirtuin 3 and ferroptosis. Sirt3 regulates iron cell metabolism by inhibiting TFR1-mediated iron uptake. Sirt3 activates NRF2 and decreases ROS production. NRF2 can act as an upstream regulator of Sirt3. Sirt3 can exhibit a pro-ferroptotic effect *via* ferritinophagy stimulation. Red arrows denote pro-ferroptotic effects and green arrows denote anti-ferroptotic effects. **(B)** Sirtuin 3 and pyroptosis. Sirt3 inhibits NLRP3 inflammasome activation and pyroptosis *via* autophagy and mitophagy stimulation. Sirt3 can promote pyroptosis *via* direct deacetylation of NLRC4 and activation of NLRC4 inflammasome. Red arrows denote pro-pyroptotic effects and green arrows denote anti-pyroptotic effects.

Ferroptosis is an iron accumulation-mediated nonapoptotic cell death, and its main features are increased cellular ROS production and the accumulation of lipid peroxide caused by iron. Sirt3 is involved in the regulation of iron transport and metabolism (Tinkov et al., 2021). Sirt3 was shown to modulate iron regulatory protein 1 (IRP1) activity, resulting in downregulation of transferrin receptor 1 (TfR1) expression. Sirt3-knockout cells have aberrant iron metabolism, which provides a higher growth rate (Jeong et al., 2015) (Figure 3A). On the other hand, iron overload can inhibit Sirt3 activity, leading to mitochondrial ROS accumulation and autophagy, which causes bone marrow damage (Zhou et al., 2021). Sirt3 activation plays a protective role in iron-overloaded liver cells *via* the Wnt/β-catenin pathway (Mandala et al., 2021). These findings shows that Sirt3 can control cell death mediated by iron overload.

An important factor that regulates iron homeostasis and oxidative stress is the transcriptional factor Nrf2. It can act as an upstream regulator of Sirt3 in neuronal cells (Gao et al., 2018). On the other hand, Sirt3 can activate Nrf2 itself and inhibit ferroptosis (Wang et al., 2022). Consistently with these data, Sirt3 was shown to decrease ROS levels and suppress glutamate-induced ferroptosis in oligodendrocytes (Novgorodov et al., 2018). Ferroptosis can be mediated by p53, which connects this cell death type to apoptosis and necroptosis (Yamada and Yoshida, 2019). Sirt3 can inhibit p53-mediated ferroptosis, protecting human cancer cells from the stress caused by ROS accumulation (Jin et al., 2021).

However, in gallbladder cancer cell lines, Sirt3 showed a pro-ferroptotic effect. It inhibited AKT-dependent mitochondrial metabolism and the epithelial-mesenchymal transition, leading to ferroptosis and tumor suppression (Liu et al., 2021b). Ferroptosis can be promoted by autophagy *via* ferritin removal (Hou et al., 2016). Increased Sirt3 expression contributed to classical ferroptotic events and autophagy activation, whereas Sirt3 silencing led to resistance to both ferroptosis and autophagy. In addition, autophagy inhibition impaired Sirt3-enhanced ferroptosis. By contrast, autophagy induction acted synergistically with Sirt3. Based on mechanistic investigations, Sirt3 depletion inhibited activation of the AMPK-mTOR pathway and enhanced the glutathione peroxidase 4 (GPX4) level, thereby suppressing autophagy and ferroptosis (Han et al., 2020). This finding reveals the involvement of Sirt3 in crosstalk between autophagy and ferroptosis.

### Sirtuin 3 and pyroptosis

Pyroptosis is an innate immunity-related type of PCD. It includes a specific type of chromatin condensation, formation of pores on the plasma membrane by gasdermin protein family members, and cell swelling (Galluzzi et al., 2018). Often, it is triggered by nucleotide-binding oligomerization domain-like receptor (NLR)-mediated inflammasome formation and inflammatory caspase activation, mainly of caspase 1. The most important NLR family member implicated in pyroptosis is NLR family pyrin domain-containing protein 3 (NLRP3).

Autophagy acts as a negative modulator of pyroptosis by eliminating essential components of this process, including NLRP3 (Guo et al., 2021). Sirt3, as an autophagy regulator, plays an important role in pyroptosis inhibition (Figure 3B). Thus, Sirt3 loss in macrophages compromised autophagy, and was followed by NLRP3 inflammasome activation and vascular metabolic inflammation (Liu et al., 2018). Similarly, in THP-1 macrophages, pyroptosis inhibition *via* electrical stimulation was accompanied by Sirt3 upregulation, autophagy activation, and attenuation of the ROS content (Cong et al., 2020). Interestingly, in these experiments, direct deacetylation of ATG5 by Sirt3 was observed.

Mitophagy also usually inhibits pyroptosis (Li et al., 2021). Sirt3-mediated mitophagy was shown to attenuate NLRP3-inflammasome activation in the hippocampus (Yu et al., 2020). Increased Sirt3 activity induced by melatonin promoted mitophagy *via* the FOXO3a/Parkin pathway and had no effect on protein expression. This led to ROS scavenging and NLRP3 inflammasome inhibition (Ma et al., 2018).

Similarly, Sirt3 regulation of necroptosis was shown to interact with pyroptosis. Thus, necroptosis induced by Sirt3 deficiency was accompanied by NLRP3-mediated inflammation, ROS production, and cardiomyocyte death in diabetic mice, which suggested a possible switch to pyroptosis (Song et al., 2021). Taken together, these data indicate that Sirt3 is a negative regulator of NLRP3 pyroptosis. On the other hand, in primary peritoneal macrophages, Sirt3 deficiency had no significant effect on NLRP3 inflammasome activation. Interestingly, in this study, Sirt3 was shown to directly deacetylate another inflammasome component, NLR domain-containing protein 4 (NLRC4), to promote pyroptosis (Guan et al., 2021).

## Concluding remarks

In conclusion, the available data show that Sirt3 plays a dual role in carcinogenesis. It can act as a tumor suppressor or promoter, depending on the cell and tumor type, cellular homeostasis, and sensitivity to cell death stimuli. Cancer cells are characterized by higher ROS levels than normal cells, and this factor confers advantages in terms of tumor promotion and progression. However, the effects of some anticancer drugs are based on their ability to stimulate ROS production, particularly upon targeting of mitochondrial respiratory complexes, to reach toxic levels, causing cell death and thus overcoming treatment resistance. Silencing Sirt3 under these circumstances provides an additional advantage in terms of cell death stimulation. Targeting Sirt3 can evoke distinct consequences depending on the combination of multiple parameters, including the significance of ROS for proliferation or cell death induction, the role of HIF1 in tumorigenesis, the expression level of pro- and antiapoptotic proteins, the predominant mode of OMM permeabilization, et cetera. Thus, Sirt3 silencing seems to be beneficial in oral and esophageal carcinomas, where it protects cells from death, while in lung, colon, breast carcinomas the role of Sirt3 is not clear, and its suppression could have protumor effect. The role of each parameter and their interaction in specific cases are still to find out.

These changes should be considered when searching for antitumor strategies based on Sirt3 targeting. Sirt3 also facilitates autophagy and mitophagy, processes that may both suppress tumor development and protect tumor cells from death, thus supporting tumor growth. Besides apoptosis and autophagy, Sirt3 modulates other cell death types such pyroptosis, necroptosis, anoikis, and ferroptosis. Its implication in these processes is rather complicated. Sirt3 usually acts as a negative regulator of necroptosis, RIP-dependent anoikis, ferroptosis, and pyroptosis, mainly through its antioxidant activity. Nevertheless, Sirt3-mediated deacetylation of specific proteins, such as p53 in necroptosis or NLRC4 in pyroptosis, makes it responsible for pro-death effects. Sirt3-mediated autophagy plays a dual role in cell death regulation. It was shown to eliminate important components of the cell death pathways, promoting (ferroptosis) or inhibiting them (pyroptosis). Therefore, investigating possible mechanisms of the activation of the Sirt3 tumor suppressor function is an important task in medical oncology.
